# Altered Cortical Thickness-Based Individualized Structural Covariance Networks in Patients with Schizophrenia and Bipolar Disorder

**DOI:** 10.3390/jcm9061846

**Published:** 2020-06-13

**Authors:** Sungkean Kim, Yong-Wook Kim, Hyeonjin Jeon, Chang-Hwan Im, Seung-Hwan Lee

**Affiliations:** 1 J. Crayton Pruitt Family Department of Biomedical Engineering, University of Florida, Gainesville, FL 32611, USA; sdm04sdm@hanmail.net; 2 Department of Biomedical Engineering, Hanyang University, Seoul 04763, Korea; kim3863215@naver.com (Y.-W.K.); ich@hanyang.ac.kr (C.-H.I.); 3 Clinical Emotion and Cognition Research Laboratory, Inje University, Goyang 411-706, Korea; albpsybm@gmail.com; 4 Department of Psychiatry, Ilsan Paik Hospital, College of Medicine, Inje University, Juhwa-ro 170, Ilsanseo-Gu, Goyang 411-706, Korea

**Keywords:** individualized structural covariance network, graph theory, cortical thickness, schizophrenia, bipolar disorder

## Abstract

Structural covariance is described as coordinated variation in brain morphological features, such as cortical thickness and volume, among brain structures functionally or anatomically interconnected to one another. Structural covariance networks, based on graph theory, have been studied in mental disorders. This analysis can help in understanding the brain mechanisms of schizophrenia and bipolar disorder. We investigated cortical thickness-based individualized structural covariance networks in patients with schizophrenia and bipolar disorder. T1-weighted magnetic resonance images were obtained from 39 patients with schizophrenia, 37 patients with bipolar disorder type I, and 32 healthy controls, and cortical thickness was analyzed via a surface-based morphometry analysis. The structural covariance of cortical thickness was calculated at the individual level, and covariance networks were analyzed based on graph theoretical indices: strength, clustering coefficient (CC), path length (PL) and efficiency. At the global level, both patient groups showed decreased strength, CC and efficiency, and increased PL, compared to healthy controls. In bipolar disorder, we found intermediate network measures among the groups. At the nodal level, schizophrenia patients showed decreased CCs in the left suborbital sulcus and the right superior frontal sulcus, compared to bipolar disorder patients. In addition, patient groups showed decreased CCs in the right insular cortex and the left superior occipital gyrus. Global-level network indices, including strength, CCs and efficiency, positively correlated, while PL negatively correlated, with the positive symptoms of the Positive and Negative Syndrome Scale for patients with schizophrenia. The nodal-level CC of the right insular cortex positively correlated with the positive symptoms of schizophrenia, while that of the left superior occipital gyrus positively correlated with the Young Mania Rating Scale scores for bipolar disorder. Altered cortical structural networks were revealed in patients, and particularly, the prefrontal regions were more altered in schizophrenia. Furthermore, altered cortical structural networks in both patient groups correlated with core pathological symptoms, indicating that the insular cortex is more vulnerable in schizophrenia, and the superior occipital gyrus is more vulnerable in bipolar disorder. Our individualized structural covariance network indices might be promising biomarkers for the evaluation of patients with schizophrenia and bipolar disorder.

## 1. Introduction

Schizophrenia and bipolar disorder are both major psychiatric disorders. Schizophrenia and bipolar disorder have both similarities and differences in terms of psychological and neuronal levels. In addition, they have overlapping symptoms [1,2]. However, the pathologies of these two diseases have not yet been revealed [1,3]. Therefore, studies that could help in understanding the pathologies of these two diseases are needed.

Magnetic resonance imaging (MRI) studies have consistently reported structural brain abnormalities in patients with schizophrenia. The most frequently reported results are gray matter volume reductions and cortical thinning in the frontal and temporal lobes [4,5,6,7,8,9]. For bipolar disorder, research results have been inconclusive, but meta-analyses reveal gray matter reductions in the anterior cingulate and fronto-insular regions [10,11]. Several studies show that structural brain abnormalities in the frontal lobe are overlapping biological features of schizophrenia and bipolar disorder [9,12,13].

Structural covariance can be defined as coordinated variation in brain morphological measurements, such as cortical thickness and volume, among brain structures functionally or anatomically interlinked with one another [14]. Compared to conventional regional analysis, recent studies have revealed that intracortical similarities, based on coordinated variations in gray matter morphology, can offer evidence for structural brain connectivity [15,16,17]. The high phenotypic covariance of brain morphology across brain areas can be interpreted as evidence advocating the existence of coordinated maturational, neurodevelopmental, and evolutionary processes in the brain [18,19,20]. Recently, a number of MRI and/or positron emission tomography studies have investigated structural covariance networks, based on graph theory [14,21,22,23,24,25,26,27]. Graph theory has been introduced as a method to construct human brain networks. Brain networks based on graph theoretical approaches could help to understand the brain mechanisms of psychiatric disorders, including schizophrenia and bipolar disorder. Previous studies have demonstrated widespread network inefficiency with abnormal information propagation, indicating disturbed integration between brain regions, in schizophrenia [22,25]. In addition, node centrality (assessing the relative importance of a node) alterations have been found, mainly in prefrontal regions and less consistently in parietal and temporal regions, in schizophrenia [28]. However, only one study investigated the patients with bipolar disorder, and showed no difference in structural covariance networks when compared to healthy controls [29].

While these studies present significant advances in the investigation of anatomical connectivity, they mainly depended on group-level structural correlations of cortical morphology [14,21,22,23,24,25,26,27]. These group-level structural covariance networks offer a statistical framework for investigating synchronized morphological alterations in brain regions across a group of subjects. However, it remains uncertain how an individual-level structural covariance network might be constructed. In fact, the morphological alterations of cortical gray matter differ considerably at the individual level [17,30]. The construction of structural covariance networks at the individual level using z-scores, with one individual having one covariance matrix, could enable the analysis of individualized structural covariance, which might well reflect individual network alterations based on morphological alterations, such as disease-related cortical changes. This could, furthermore, allow statistical analyses of the theoretical characteristics of individualized structural covariance networks, which is a challenging task to undertake using group-level structural covariance networks. Particularly, correlation analyses between individualized structural covariance networks and clinical scales in psychiatric disorders could help to understand their pathophysiology. No study so far has investigated and compared altered structural covariance networks at the individual level, using cortical thickness in patients with schizophrenia and bipolar disorder.

There are several reasons that make cortical thickness-based structural networks particularly noteworthy [14,21]. Even though cortical thickness is affected by the extracellular space, it is a biologically important measure that could reflect the density, size and disposition of cells, including neurons, neuroglia and nerve fibers [31]. Many network results based on cortical thickness are in line with cortical functional network results from functional MRIs performed during resting state, indicating that structural and functional networks are topologically isomorphic [21,32,33,34,35,36].

In the present study, we investigated and compared cortical thickness-based individualized structural covariance networks in patients with schizophrenia and bipolar disorder, using a weighted network analysis. This analysis allowed us to assess structural network alterations in not only global network patterns, but also in specific local cortical regions. Furthermore, we explored the relationships between structural covariance network indices and psychological measures, which would help in comprehending the pathologies of schizophrenia and bipolar disorder. We hypothesized that patient groups would reveal abnormal network topological organization, at both the global (whole brain) and nodal (each region) levels. More specifically, we predicted lower clustering and higher integration in the patients compared to healthy controls. In addition, we hypothesized that the altered cortical network indices would be significantly associated with psychiatric symptom scales, reflecting the core pathological symptoms of the patients.

## 2. Materials and Methods

### 2.1. Participants

A total of 108 subjects between the ages of 20 and 64 years participated in this study. The subjects included patients with schizophrenia (*n* = 39, age: 43.62 ± 11.11 (range: 21–60)) and bipolar disorder (*n* = 37, age: 40.24 ± 12.72 (range: 20–63)) as well as healthy controls (*n* = 32, age: 44.59 ± 12.51 (range: 23–64)). All patients with bipolar disorder were diagnosed with type I. All patients were assessed for Axis I [37] and II [38] disorders, based on the Structured Clinical Interview for the Diagnostic and Statistical Manual of Mental Disorders, 4th edition (SCID), by a board-certified psychiatrist. No patient had a lifetime history of alcohol or drug abuse, mental retardation, central nervous system disease or head injury with loss of consciousness. Patients with schizophrenia were being treated with atypical antipsychotics, and patients with bipolar disorder with mood-stabilizing agents (lithium, topiramate, lamotrigine and sodium valproate), with or without atypical antipsychotics. All recruited patients were under treatment at Inje University Ilsan Paik Hospital. The 32 healthy controls were recruited from the local community through newspapers and flyers. An initial screening interview excluded subjects with any identifiable neurological disorder or head injury, or any personal or family history of psychiatric illness. After the initial screening, potential healthy controls were interviewed using the SCID for Axis I [37] (schizophrenia and bipolar disorder) and Axis II [38] (schizotypal and schizoid personality disorder) disorders and were excluded if they had any of these disorders. All subjects signed a written informed consent form approved by the Institutional Review Board of Inje University Ilsan Paik Hospital (2015-07-23).

### 2.2. Symptomatic and Psychological Measures

Psychiatric symptoms were evaluated using the Positive and Negative Syndrome Scale (PANSS) for schizophrenia [39] and the Young Mania Rating Scale (YMRS) for bipolar disorder [40]. To evaluate neurocognition, a verbal fluency test [41] and the Korean version of the Auditory Verbal Learning Test (K-AVLT) [42] were applied. In the verbal fluency test, the participants named as many animals as possible within 60 s. This task evaluates verbal production and semantic memory abilities [41]. The K-AVLT, which is included in the Rey–Kim Memory Test [42], is a verbal memory test that consists of five “immediate recall” trials (trials 1–5), plus “delayed recall” and “delayed recognition” trials. The immediate recall score is the sum of the words (trials 1–5) recalled correctly. The delayed recall score indicates the number of words recalled correctly after a delay period of 20 min. The delayed recognition score indicates the words correctly chosen from the original list (15 words) read out by the examiner, presented in a list of 50 words after the delayed recall trials. In addition, the premorbid IQ was measured using the information test from the Korean Wechsler Adult Intelligence Scale (K-WAIS-IV), age and education year [43].

### 2.3. MRI Acquisition and SBM

MRI was conducted using a 1.5 T scanner (Magneton Avanto, Siemens, Erlangen, Germany). Head motion was minimized using restraining foam pads provided by the manufacturer. High-resolution T1-weighted MRI images were obtained with the following acquisition parameters: a 227 × 384 acquisition matrix, a 210 × 250 field of view, a voxel size of 0.9 × 0.7 × 1.2, a total of 87,168 voxels, a TE of 3.42 ms, a TR of 1900 ms, 1.2-mm slice thickness, and a flip angle of 15°.

All images were examined visually for motion or other artifacts before and after preprocessing. A surface-based morphometry (SBM) analysis was performed using CAT12 (http://dbm.neuro.uni-jena.de/cat/) implemented in SPM12 (Wellcome Department of Cognitive Neurology, London, UK). SPM12 tissue probability maps were used for the initial spatial registration. The structural T1 images were registered to an ICBM East Asian template and normalized using the DARTEL algorithm [44]. The images were then segmented into gray matter, white matter and cerebrospinal fluid [45]. Jacobian-transformed tissue probability maps were used to modulate images. The projection-based thickness method was applied to the SBM analysis to estimate cortical thickness for the left and right hemisphere [46]. The cortical thickness of the regions was extracted using the Destrieux atlas, which contains 74 cortical areas in each hemisphere [47]. Segmentation was automatically conducted using probabilistic methods [48].

### 2.4. Cortical Thickness-Based Individualized Structural Covariance Network Analysis

First, in order to calculate the cortical thickness-based structural covariance at the individual level, the cortical thickness values of 148 regions per participant were normalized using the mean and standard deviation values of the 148 regions for each participant [49]. Then, the cortical thickness-based individualized structural covariance values for each pair of the 148 regions were calculated using the following equation [50,51]:

individualized structural covariance (i, j) = 1/e((*n*(i) − *n*(j))^2^) (1)

where *n*(i) and *n*(j) represent the z-scored values of cortical thickness of each participant, based on the mean and standard deviation values across all healthy controls (*n* = 32) for each region, respectively. Finally, a 148 × 148 matrix including a total of 10,878 (_148_*C*_2_, each pair of 148 regions) cortical thickness-based individualized structural covariances per participant was generated for the subsequent graph theoretical analyses.

In the present study, the weighted network analysis was conducted based on graph theory [52,53]. The use of weighted networks is not only free from ambiguity in determining the threshold values, but can also preserve the unique traits of the original network without distortion. A network is composed of several nodes that are connected to each other at their edges. In this study, we selected representative network measures. Four different global-level weighted network indices were evaluated exploratively given the number of possible network indices that could have been assessed. First, “strength” refers to the degree of connection strength in the network. It is estimated by summing up the weights of links connecting brain regions. A higher strength value means that the whole brain is strongly connected. Second, the “clustering coefficient” (CC) indicates the degree to which a node clusters with its neighboring nodes. An increased CC indicates that a network is well segregated between the relevant brain regions. The CC was calculated for the whole network. Third, “path length” (PL) indicates the sum of lengths between two nodes within the network, which is related to the speed of information processing. A shortened PL indicates a well-integrated network. Fourth, “efficiency” refers to the effectiveness of information processing in the brain; high efficiency indicates rapid information propagation in the network. Additionally, the weighted nodal CC was evaluated for each node. Network analyses were performed using MATLAB R2016b (MathWorks, Natick, MA, USA).

### 2.5. Statistical Analysis

Chi-squared tests and one-way analysis of variance (ANOVA) were used to examine differences in demographic variables and psychological scores among the three groups. In addition, a multivariate ANOVA (MANOVA) was conducted to compare the cortical network characteristics at the global level among the three groups, with premorbid IQ as a covariate. *p*-values were adjusted with the false discovery rate (FDR) method to control multiple comparisons [54]. The identical analysis was conducted at the nodal level, followed by the FDR method. The variables showing significant differences among the three groups were further analyzed using post-hoc pairwise comparisons, using least significant difference (LSD). Effect sizes were expressed as partial eta squared (η^2^). A partial Pearson’s correlation analysis was conducted between network indices and psychological measures, with a 5000-bootstrap resampling technique to correct for multiple correlations in each group. The bootstrap test is weaker than the Bonferroni test at solving the multiple-comparison problem; however, the robustness and stability of the bootstrap test have been documented in various previous studies [55,56,57]. Moreover, the bootstrap test has been widely used in MRI analysis [58,59,60]. For the patient groups, the potential effects of medication (equivalent doses of chlorpromazine and sodium valproate) [61] and duration of illness were considered as covariates. The significance level was set at *p* < 0.05 (two-tailed). Statistical analyses were performed using SPSS 21 (SPSS, Inc., Chicago, IL, USA).

## 3. Results

### 3.1. Demographic and Psychological Characteristics

Table 1 shows the comparison of demographic and psychological characteristics among the schizophrenia, bipolar disorder and healthy control groups. Premorbid IQ differed significantly among the three groups; healthy controls showed a significantly higher premorbid IQ than patients with schizophrenia or bipolar disorder (100.60 ± 10.17 vs. 97.73 ± 8.19 vs. 107.03 ± 9.38, *p* < 0.001). The verbal fluency score was significantly lower in both patient groups than in healthy controls (15.21 ± 4.95 vs. 14.57 ± 5.37 vs. 18.90 ± 5.96, *p* = 0.003). Furthermore, the K-AVLT-trial 5 score was significantly lower in the schizophrenia than in the bipolar disorder and healthy control groups (8.77 ± 2.77 vs. 10.22 ± 2.84 vs. 11.50 ± 1.78, *p* < 0.001).

### 3.2. Global- and Nodal-Level Differences in Cortical Functional Networks

Table 2 shows the comparison of global-level indices, including strength, CC, PL and efficiency, among the schizophrenia, bipolar disorder and healthy control groups. Strength, CC and efficiency were significantly decreased in the patients with schizophrenia and bipolar disorder, as compared to the healthy controls (strength: 63.19 ± 6.18 vs. 64.61 ± 7.10 vs. 68.81 ± 5.56, *p* = 0.001; CC: 0.31 ± 0.05 vs. 0.32 ± 0.06 vs. 0.36 ± 0.05, *p* = 0.001; efficiency: 0.50 ± 0.03 vs. 0.51 ± 0.04 vs. 0.53 ± 0.03, *p* = 0.001). PL, in contrast, was significantly increased in patients with schizophrenia and bipolar disorder (3.08 ± 0.34 vs. 3.02 ± 0.43 vs. 2.74 ± 0.24, *p* < 0.001).

Based on the significant differences in global CCs among the three groups, we decided to examine possible differences at the local level. The nodal CCs among the three groups significantly differed in four nodes (left suborbital sulcus: 0.25 ± 0.13 vs. 0.34 ± 0.12 vs. 0.37 ± 0.13, *p* = 0.037; right superior frontal sulcus: 0.24 ± 0.15 vs. 0.31 ± 0.13 vs. 0.36 ± 0.13, *p* = 0.037; right long insular gyrus and central insular sulcus: 0.23 ± 0.13 vs. 0.26 ± 0.15 vs. 0.36 ± 0.11, *p* < 0.001; left superior occipital gyrus: 0.24 ± 0.16 vs. 0.24 ± 0.15 vs. 0.36 ± 0.13, *p* < 0.001). The nodal CCs of the schizophrenia group were significantly decreased in the left suborbital sulcus and right superior frontal sulcus, as compared to the bipolar disorder group and healthy controls. The nodal CCs of the schizophrenia and bipolar disorder groups were significantly decreased in the right long insular gyrus and central insular sulcus, as well as in the left superior occipital gyrus, as compared to the healthy controls (Table 3).

### 3.3. Correlations between Network Indices and Psychological Characteristics

**Figure 1:**
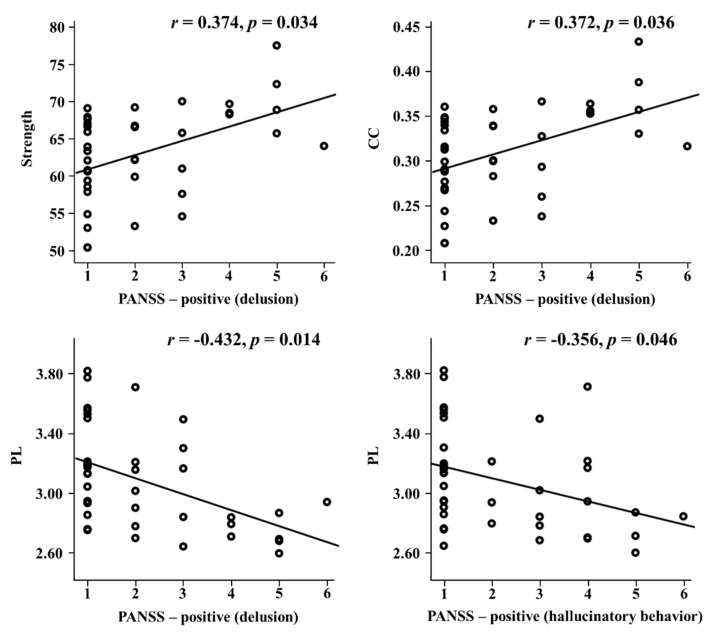
Correlations of global network indices with PANSS positive subscales in schizophrenia. PANSS: positive and negative syndrome scale; CC: clustering coefficient; PL: path length.

The relationships between the network indices and psychological measures were investigated at the global and nodal level. At the global level, significant relationships were only observed in the schizophrenia group. PL significantly correlated with PANSS positive symptoms (r = −0.378, *p* = 0.033), and four global network indices were associated with two PANSS positive subscales: delusion (strength: r = 0.376, *p* = 0.034; CC: r = 0.372, *p* = 0.036; PL: r = −0.432, *p* = 0.014; efficiency: r = 0.380, *p* = 0.032) and suspiciousness/persecution (strength: r = 0.386, *p* = 0.029; CC: r = 0.390, *p* = 0.027; PL: r = −0.439, *p* = 0.012; efficiency: r = 0.385, *p* = 0.030). In addition, PL significantly correlated with the PANSS positive subscale of hallucinatory behavior (r = −0.356, *p* = 0.046) (Figure 1).

**Figure 2:**
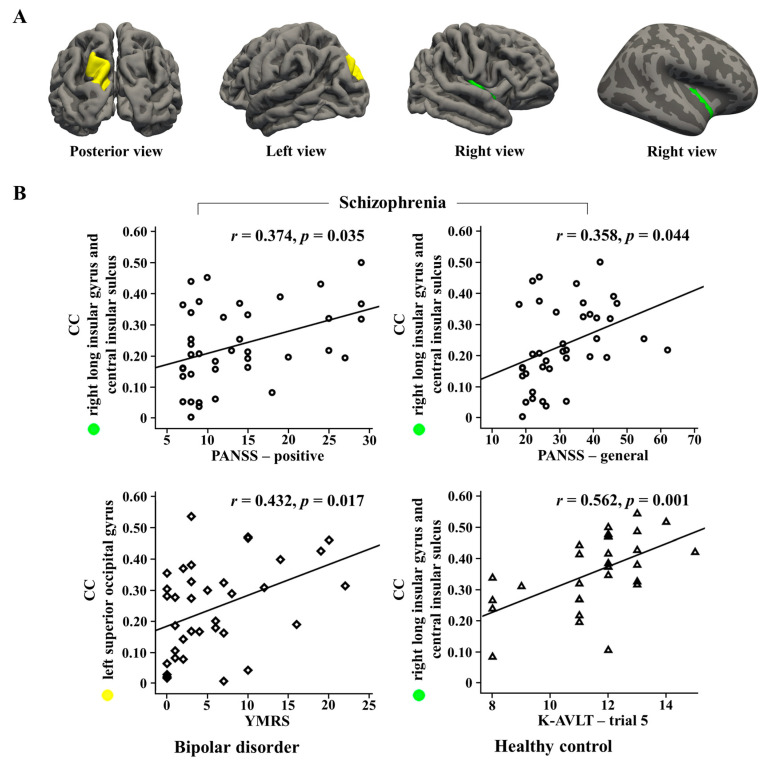
(**A**) Brain regions of right long insular gyrus and central insular sulcus, and left superior occipital gyrus (**B**) Correlations of nodal clustering coefficients (CCs) in two regions with psychological measures in each group. PANSS: positive and negative syndrome scale; YMRS: Young Mania Rating Scale; K-AVLT: Korean Auditory Verbal Learning Test.

At the nodal level, the nodal CC in the right long insular gyrus and the central insular sulcus significantly correlated with PANSS positive (r = 0.374, *p* = 0.035) and general symptoms (r = 0.358, *p* = 0.044) in the schizophrenia group. Moreover, the nodal CC was significantly associated with the PANSS positive subscales of conceptual disorganization (r = 0.365, *p* = 0.040), suspiciousness/persecution (r = 0.412, *p* = 0.019) and hostility (r = 0.602, *p* < 0.001) in the schizophrenia group. In the bipolar disorder group, the nodal CC in the left superior occipital gyrus was significantly associated with the YMRS (r = 0.432, *p* = 0.017). In healthy controls, the nodal CC in the right long insular gyrus and the central insular sulcus significantly correlated with the K-AVLT-trial 5 (r = 0.562, *p* = 0.001) (Figure 2).

## 4. Discussion

This study evaluated cortical structural networks derived from cortical thickness-based individualized structural covariance in patients with schizophrenia and bipolar disorder, as compared to healthy controls. First, at the global level, strength, CC and efficiency were significantly decreased, while PL was increased in both patient groups. Second, at the nodal level, the CCs were significantly decreased in the left suborbital sulcus and right superior frontal sulcus in patients with schizophrenia, as compared to patients with bipolar disorder. The CCs in the right long insular gyrus, the central insular sulcus, and the left superior occipital gyrus were significantly decreased in both patient groups. Third, the global-level network indices, including strength, CC and efficiency, were significantly positively correlated, while PL was negatively correlated, with positive PANSS symptoms in patients with schizophrenia. Fourth, the nodal-level CC of the right long insular gyrus and the central insular sulcus was significantly positively correlated with PANSS positive and general symptoms in the schizophrenia group, while that of the left superior occipital gyrus was significantly positively correlated with YMRS scores in the bipolar disorder group.

Strength, CC and efficiency were significantly decreased in both patient groups, as compared to healthy controls. PL, in contrast, was significantly increased in the patient groups. Structural network studies using diffusion tensor imaging have reported increased PL or decreased efficiency in patients with schizophrenia [62,63]. In bipolar disorder, structural brain networks, from diffusion tensor imaging, exhibited lower CCs and efficiency and longer PL [64,65]. Only one previous study examined cortical structural networks using cortical thickness in patients with schizophrenia [25], and reported increased CCs and PL in patients with schizophrenia. The difference between our results and the earlier study may be caused by differences in demographic characteristics and symptom severity, such as, for example, older age, longer duration of illness and higher PANSS total and negative symptoms in our participants. Our findings support the previous results, in that our patients with schizophrenia and bipolar disorder showed abnormal global topological organization of cortical structural networks. Furthermore, our findings revealed intermediate network measures of patients with bipolar disorder, between those found in patients with schizophrenia and those in healthy controls, although two patient groups did not show significantly different network measures. It might imply a possibility that the networks of the patients with schizophrenia were more altered than those of the patients with bipolar disorder.

Interestingly, the nodal CCs were significantly decreased in the left suborbital sulcus and right superior frontal sulcus in patients with schizophrenia, as compared to those with bipolar disorder and the healthy controls. The suborbital sulcus is a medial sub-region of the orbitofrontal cortex. The medial orbitofrontal cortex is involved in sensory integration, social cognition and metacognition [66]. Brain imaging studies have revealed abnormalities or dysfunctions of this region in schizophrenia patients [66,67,68]. In addition, previous studies showed an association between cortical thinning of the left medial orbitofrontal cortex and negative symptom severity, underlining the importance of this region in impaired executive and motivational functioning in schizophrenia patients [69,70]. The superior frontal region has been known to be associated with cognitive control, including set-switching [71], working memory [72] and complex problem solving [73]. The cortical thickness of the superior frontal region was significantly decreased in patients with schizophrenia, as compared to patients with bipolar I disorder [9] and healthy controls [74,75]. In addition, an functional MRI (fMRI) study investigating default mode network at rest found an activation difference in the superior frontal region between schizophrenia and bipolar disorder [76]. According to the preexisting notion, our findings suggest structural abnormalities of the suborbital sulcus and superior frontal sulcus belonging to the prefrontal cortex, which might imply that cognitive functions are vulnerable in patients with schizophrenia, compared to those with bipolar disorder and healthy controls.

The nodal CCs of patients with schizophrenia and bipolar disorder were significantly decreased in the right insular cortex, as compared to those of healthy controls. There is converging evidence of functional and structural abnormalities in the insular cortex in schizophrenia and bipolar disorder [11,77,78,79,80,81,82,83,84,85]. The insular cortex is in charge of sensory integration, language processing and emotional information integration [86,87,88,89,90]. Particularly in schizophrenia, strength, CC, PL and efficiency showed significant correlations with PANSS positive symptoms. In addition, the nodal CC of the right insular cortex was significantly positively correlated with PANSS positive symptoms. Previous studies have demonstrated a correlation between the insular cortex and positive symptoms in schizophrenia [78,86,91,92]. Our findings suggest a strong structural abnormality of the insula, which is related to sensory integration and language functions, in patients with schizophrenia.

In healthy controls, the nodal CC in the right insular cortex showed a significant positive correlation with immediate verbal memory. A study using positron emission tomography showed a correlation between cerebral blood flow in the insular cortex and verbal memory tasks in healthy controls [93]. Our results suggest that the nodal CC of the insular cortex, a language processing-related region, correlates with verbal memory in healthy controls.

Furthermore, the nodal CCs of patients with schizophrenia and bipolar disorder were significantly decreased in the left superior occipital gyrus compared to healthy controls. Chan et al. [94] found an association between longer durations of illness and gray matter reductions in the superior occipital gyrus in patients with schizophrenia. In addition, resting-state fMRI studies showed reduced spontaneous neural activity in the superior occipital gyrus in patients with schizophrenia [95] or bipolar disorder [96]. Another study, using structural and diffusion MRI, showed reduced CC and efficiency in this region in patients with bipolar disorder [97]. We found here that the nodal CC of the left superior occipital gyrus was significantly positively correlated with YMRS scores in the bipolar disorder group. The superior occipital gyrus might be associated with emotional processing, as the activation of this region has been reported during tasks related to emotion recognition [98,99,100]. However, the relationship between the superior occipital gyrus and psychiatric symptoms has rarely been investigated in bipolar disorder.

The patient groups showed positive correlations between nodal CCs and psychiatric symptoms, including PANSS positive symptoms and YMRS scores. These results could be explained in two ways. First, the symptoms of the patients, such as auditory hallucinations, may have contributed to, or might have been affected by, the inefficient networks with excessive clustering among regions [101,102,103]. Second, some patients may have recovered their brain structure as a result of the antipsychotic treatment [104], even if their psychotic symptoms are still present or worsening [105].

Taken together, our study revealed that the nodal CCs of the suborbital sulcus and superior frontal sulcus (cognition-related regions) were decreased in patients with schizophrenia, as compared to those with bipolar disorder. Furthermore, we demonstrated that the nodal CC of the insular cortex (a cognition-related region) correlated with PANSS positive symptoms in patients with schizophrenia, while the nodal CC of the superior occipital gyrus (an emotion-related region) correlated with YMRS scores in patients with bipolar disorder, suggesting that nodal CCs might be predictable biomarkers of psychiatric symptoms.

This study has the following limitations: First, most of patients had chronic forms of both diseases, and were taking atypical antipsychotics and mood-stabilizing agents. Although we controlled the duration of illness and dosage of medication, future studies should consider the cumulative effect of medication treatment, and also include drug-naive patients. Second, the structural covariance of abnormality in the patients was based on relatively small healthy control samples. Further study needs to incorporate larger healthy control samples. Third, our hemisphere-specific findings need to be interpreted with caution. Brain regions with pathological abnormalities can vary depending on the disease’s heterogeneity, the number of patients, duration of illness, and so on. Since the patients in this study showed a relatively high female proportion, relatively long duration of illness, and relatively stabilized symptom scores, these characteristics need to be taken into account for interpretation. Therefore, our findings should be further replicated with a larger number of patients, considering various characteristics such as gender, duration of illness and symptom scores.

## 5. Conclusions

This study represents the first attempt to compare cortical structure networks between patients with schizophrenia and those with bipolar disorder using cortical thickness-based individualized structural covariance. Our results demonstrate altered cortical structural networks at both the global and the nodal level in the patient groups. Particularly, the schizophrenia patients showed lower structural clustering in prefrontal regions, including the suborbital sulcus and superior frontal sulcus, as compared to bipolar disorder patients. We also found significant correlations between altered cortical structure network states and core pathological symptoms. Our cortical structural network indices might thus be promising biomarkers for the evaluation of patients with schizophrenia and bipolar disorder. They could be used for predicting cases or partitioning cases into subgroups with similar brain abnormalities. In particular, in clinical reality, they could be utilized as a useful auxiliary means, such as in assisting with more accurate diagnosis, helping in the guidance of finding the right medication, and serving as helpful indicators for predicting treatment outcomes in the two psychiatric disorders.

## Figures and Tables

**Table 1:** Demographic characteristics of all study participants.

	Schizophrenia ^a^ (*n* = 39)	Bipolar Disorder ^b^ (*n* = 37)	Healthy Controls ^c^ (*n* = 32)	*p*	Post-Hoc (LSD)
Age (years)	43.62 ± 11.11	40.24 ± 12.72	44.59 ± 12.51	0.286	
Sex				0.182	
Male	17 (43.6)	10 (27.0)	15 (46.9)		
Female	22 (56.4)	27 (73.0)	17 (53.1)		
Premorbid IQ	100.60 ± 10.17	97.73 ± 8.19	107.03 ± 9.38	<0.001	a < c, b < c
Education (years)	13.28 ± 2.68	12.73 ± 2.70	13.59 ± 3.77	0.490	
Number of hospitalizations	3.28 ± 4.09	2.47 ± 2.63		0.316	
Duration of illness (years)	13.46 ± 9.54	9.50 ± 6.90		0.053	
Onset age (years)	29.23 ± 10.71	30.94 ± 12.93		0.551	
Dosage of medication (CPZ equivalent, mg)	395.90 ± 480.77	253.53 ± 316.99			
Dosage of medication (equivalent to sodium valproate dose, mg)	101.28 ± 250.39	790.49 ± 530.04			
PANSS					
Positive	13.74 ± 7.16	8.95 ± 2.05			
Delusion	2.21 ± 1.51	1.16 ± 0.44			
Conceptual disorganization	2.23 ± 1.51	1.19 ± 0.52			
Hallucinatory behavior	2.23 ± 1.55	1.08 ± 0.36			
Excitement	1.54 ± 0.94	1.86 ± 0.86			
Grandiosity	1.49 ± 0.97	1.35 ± 0.72			
Suspiciousness/persecution	2.46 ± 1.39	1.57 ± 0.65			
Hostility	1.59 ± 0.85	1.19 ± 0.52			
Negative	17.28 ± 6.76	9.03 ± 2.69			
General	31.28 ± 10.91	24.03 ± 6.03			
Total	62.31 ± 22.22	42.00 ± 8.86			
YMRS		5.78 ± 3.00			
Verbal fluency	15.21 ± 4.95	14.57 ± 5.37	18.90 ± 5.96	0.003	a < c, b < c
K-AVLT-trial 5	8.77 ± 2.77	10.22 ± 2.84	11.50 ± 1.78	<0.001	a < b, a < c, b < c

LSD: least significant difference; IQ: intelligence quotient; CPZ: chlorpromazine; PANSS: positive and negative syndrome scale; YMRS: Young Mania Rating Scale; K-AVLT: Korean Auditory Verbal Learning Test; a: schizophrenia; b: bipolar disorder; c: healthy controls.

**Table 2:** Mean and standard deviation values of global network indices, including strength, clustering coefficient (CC), path length (PL) and efficiency, for the schizophrenia, bipolar disorder and healthy control groups.

	Schizophrenia ^a^ (*n* = 39)	Bipolar Disorder ^b^ (*n* = 37)	Healthy Controls ^c^ (*n* = 32)	Effect Size (η^2^)	*p* *	Post-Hoc (LSD)
Strength	63.19 ± 6.18	64.61 ± 7.10	68.81 ± 5.56	0.135	0.001	a < c, b < c
CC	0.31 ± 0.05	0.32 ± 0.06	0.36 ± 0.05	0.132	0.001	a < c, b < c
PL	3.08 ± 0.34	3.02 ± 0.43	2.74 ± 0.24	0.171	<0.001	a > c, b > c
Efficiency	0.50 ± 0.03	0.51 ± 0.04	0.53 ± 0.03	0.137	0.001	a < c, b < c

a: schizophrenia; b: bipolar disorder; c: healthy controls. * The *p*-value was adjusted via false discovery rate.

**Table 3:** Mean and standard deviation values of clustering coefficients at the nodal level for the schizophrenia, bipolar disorder and healthy control groups.

	Schizophrenia ^a^ (*n* = 39)	Bipolar Disorder ^b^ (*n* = 37)	Healthy Controls ^c^ (*n* = 32)	Effect Size (η^2^)	p *	Post-Hoc (LSD)
Left suborbital sulcus	0.25 ± 0.13	0.34 ± 0.12	0.37 ± 0.13	0.135	0.037	a < b, a < c
Right superior frontal sulcus	0.24 ± 0.15	0.31 ± 0.13	0.36 ± 0.13	0.130	0.037	a < b, a < c
Right long insular gyrus and central insular sulcus	0.23 ± 0.13	0.26 ± 0.15	0.36 ± 0.11	0.154	<0.001	a < c, b < c
Left superior occipital gyrus	0.24 ± 0.16	0.24 ± 0.15	0.36 ± 0.13	0.175	<0.001	a < c, b < c

a: schizophrenia; b: bipolar disorder; c: healthy controls. * The *p*-value was adjusted via false discovery rate.
